# On species richness and rarefaction: size- and coverage-based techniques quantify different characteristics of richness change in biodiversity

**DOI:** 10.1007/s00285-018-1255-5

**Published:** 2018-06-27

**Authors:** Hideyasu Shimadzu

**Affiliations:** Department of Mathematical Sciences, Loughborough University, Loughborough, Leicestershire, LE11 3TU UK

**Keywords:** Coverage-based, Rarefaction, Richness, Size-based, Sampling, Species abundance distribution (SAD), 62D05, 92B10, 92D40

## Abstract

Changes in biodiversity today shape the future patterns of biodiversity. This fact underlines the importance of understanding changes in biodiversity through time and space. The number of species, known as species richness, has long been studied as a key indicator that quantifies the state of biodiversity, and standardisation techniques, called rarefaction, have also been used to undertake a fair comparison of the richness observed at different times or locations. The present study asks whether utilising different rarefaction techniques attains comparable results when investigating changes in species richness. The study framework presents the statistical nature of two commonly adopted rarefaction techniques: size-based and coverage-based rarefaction. The key finding is that the rarefied richness results calculated by these two different rarefaction methods reflect different aspects of biodiversity change, the shift in community size and/or composition. This fact illuminates that richness analyses based on different rarefaction techniques can reach different conclusions that may be contradictory. The study also investigates the mechanism creating such divergence. As such, special care is required when evaluating biodiversity change using species richness as an indicator.

## Introduction

Growing concern about biodiversity change highlights the pressing need for an improved understanding of the nature of this change as changes in the present shape future biodiversity patterns. However, the recent continuous debate on this topic (Vellend et al. 2013; Cardinale 2014; Dornelas et al. 2014a, b; Elahi et al. 2015; Gonzalez et al. 2016; Vellend et al. 2017, for example) to some extent indicates the degree of difficulty in quantifying biodiversity change from observed ecological data. In fact, even the popular biodiversity index on which the present study focuses—the number of species, often called species *richness*—is one of the indices that is difficult to work with, despite its familiarity, because ecological data always contain some extent of uncertainty due to their survey or sampling conditions (Shimadzu et al. 2016).

Comparing *observed* richness amongst ecological communities at different sites or times has long been recognised as an important but challenging task in quantitative ecology as the survey conditions under which data are collected may easily vary from one observation to another. The difficulty mainly stems from the fact that the observed richness increases or decreases in magnitude non-linearly with three factors: the number of individuals, the size of the survey area and the degree of survey completeness. Thus, ecologists have adopted standardisation methods known as *rarefaction* when comparing the richness of different sites or time periods (Gotelli and Colwell 2001; Chao and Jost 2012).

To date, common rarefaction techniques take one of two forms. One is a conventional approach, *size*-based rarefaction, which has a long history and traces its roots back to a benthic study concerning an assembly of *pooled* individual organisms (Sanders 1968). See Hurlbert (1971), Simberloff (1972), Heck et al. (1975) for the formal framework for this approach; Gotelli and Colwell (2001) provides a comprehensive review. The other is a relatively new technique discussed by Alroy (2010), Chao and Jost (2012), namely, *coverage*-based rarefaction. The way in which observed data are standardised to calculate *rarefied* richness distinguishes these two rarefaction techniques. A detailed comparison of these techniques will be discussed in a later section.

However, whether analyses of richness adopting different rarefaction techniques arrive at the same conclusion has rarely been examined. Much of the previous research on rarefaction techniques has focused on its merit and the inevitable downwards bias in rarefied richness. There is little theoretical investigation asking whether these two rarefaction approaches are parallel, particularly in the context of analysing richness changes over time and space. A crucial question to be asked is how the analysis results ought to be interpreted if they are contradictory, for instance, a situation where one rarefaction technique indicates an increasing or a decreasing trend but the other illustrates a flat trend that implies no changes in species richness. The present study stresses that comprehending the statistical nature of these common rarefaction techniques is a crucial step towards enhancing the knowledge of the types of biodiversity change that are currently being quantified and discussed. Further insights that underpin a better interpretation of biodiversity change as appraised by species richness are required, given that evaluating biodiversity change is becoming a matter of great importance in society.

The remainder of the paper presents new insights into the analysis of richness change, asking whether the utility of different rarefaction techniques acquires comparable results. Section 2 introduces two key concepts: marginal and conditional richness. Section 3 then describes the theoretical bases for the study framework, which establishes the formal expression of species richness given in Sect. 2. Conditional richness expresses the statistical nature of rarefaction techniques in a formal manner, specifying the rarefaction mechanism as a type of simple random sampling (Sect. 4). The current study focuses on examining the temporal trend of richness, and the gradient of conditional richness over time is then investigated for each rarefaction technique. Section 5 delineates the extent to which the trend component of richness consists of two parts: changes in community composition and abundance. The trends of rarefied richness produced by the two different rarefaction techniques reflect, in fact, ecologically different aspects of biodiversity. This finding means that richness analyses based on different rarefaction techniques can reach conflicting conclusions. A numerical simulation is performed in Sect. 6 and exhibits an agreement with the theoretical results, illuminating the mechanism that creates the divergence between these two different rarefaction techniques.

## Richness

The concept of species richness, the number of species, seems intuitive. Nevertheless, this concept is still somewhat confusing without any formal definition. To avoid unnecessary confusion, two types of richness, which have barely been distinguished in quantitative ecology, are introduced.

First, let *k* be the number of species that potentially reside in the ecological community of interest. Note that this finite number *k* is not random and fixed. Here and throughout the manuscript, as commonly used, uppercase letters denote random variables, and lowercase letters indicate the responding values, the real numbers that the random variables map into.

### Definition 1

(*Richness*) Richness, *S*, is the number of species for which at least one individual exists at a location and/or a time point. Letting $N_i$ be the number of individuals of the *i*th species, richness is defined as

1 $$\begin{aligned} S = \sum _{i=1}^k I(N_i > 0), \end{aligned}$$

where $I(\cdot )$ is the indicator function.

The number of absent species, the counterpart of the species that are present, is then described as $S_0 = \sum _{i=1}^k I(N_i = 0).$

### Definition 2

(*Conditional richness*) Conditional richness, *S*(*n*), is the number of species for which at least one individual is observed, in *a pooled sample of n individuals*. Here, $n =\sum _{i=1}^k n_i$. Conditional richness is then defined, using the indicator function $I(\cdot )$, as

2 $$\begin{aligned} S(n) = \sum _{i=1}^k I(N_i > 0 | N=n). \end{aligned}$$

Due to the natural variability within ecological communities, each species can be absent from a pooled sample, which is the case when none of the individuals of species *i* is observed, $N_i=0$. The number of unobserved species conditioning on pooled *n* individuals is therefore given as $S_0(n)=\sum _{i=1}^k I(N_i = 0 | N=n).$

Note that a key difference between richness (1) and conditional richness (2) is whether the index depends upon the total number of observed individuals, *n*. Thus, conditional richness is denoted by *S*(*n*), in contrast with richness, denoted by *S*.

## Framework

### Ecological communities

Assume that ecological communities potentially consist of a total of *k* species and that each individual is randomly distributed over a geological region, say, ${\mathcal {A}} \subset \mathbb {R}^2$. In general, space ${\mathcal {A}}$ is a plane but can also be a space defined by other auxiliary variables, such as environmental factors. The number of individuals of the *i*th species in the region, $N_i({\mathcal {A}})$, or numerical *abundance*, then follows a Poisson distribution with its mean ${\mathbb E}_{}\left[ N_i ({\mathcal {A}}) \right] = \varLambda _i ({\mathcal {A}}) = \int _{{\mathcal {A}}} \lambda _i (\varvec{x}) d\varvec{x}$, where $\lambda _i (\cdot )$ is the intensity function of species *i*, which uniquely specifies a Poisson point process (Cressie 1993; Illian et al. 2008). If the density function is location independent, *i.e.*, $\lambda _i$, it is then a homogeneous Poisson point process, and its mean becomes $\varLambda _i({\mathcal {A}}) = \lambda _i |{\mathcal {A}}|$, where the modulus sign denotes the volume (area) of space ${\mathcal {A}}$. Given a group of *k* species, the total abundance within space ${\mathcal {A}}$ is expressed as $N({\mathcal {A}}) = \sum _{i=1}^k N_i({\mathcal {A}})$, and its expectation becomes ${\mathbb E}_{}\left[ N({\mathcal {A}}) \right] = \varLambda ({\mathcal {A}}) = \sum _{i=1}^k \varLambda _i({\mathcal {A}})$. The joint distribution of species abundances, $\varvec{N}({\mathcal {A}})=(N_1({\mathcal {A}}), N_2({\mathcal {A}}), \ldots , N_k({\mathcal {A}}))$, is then given as a product of Poisson distributions for which each mean is $\varLambda _i({\mathcal {A}})$.

A longstanding goal of ecological studies is to comprehend the stochastic nature of ecological communities. Typical examples include modelling the mean abundance of each species, $\varLambda _i ({\mathcal {A}})$, and accounting for other variables with regression-type models, namely, species distribution models (SDMs: Guisan and Zimmermann 2000). These models often employ a range of statistical modelling frameworks, such as generalised linear (GLMs: McCullagh and Nelder 1989) and generalised additive (GAMs: Hastie and Tibshirani 1990) models. Recent advanced SDMs also include mixture models that cope with the heterogeneity that is often observed in ecological data (Dunstan et al. 2011, 2013).

### The distribution of observed abundances

The joint distribution of species abundances, $\varvec{N}({\mathcal {A}})=(N_1({\mathcal {A}}), N_2({\mathcal {A}}), \ldots , N_k({\mathcal {A}}))$, is expressed as a product of Poisson distributions. Conditioning on the total number of individuals $N({\mathcal {A}})=n$, in other words, fixing survey area ${\mathcal {A}}$, the conditional distribution of species abundances for pooled individuals becomes a multinomial distribution,3where $p_i= \varLambda _i ({\mathcal {A}}) / \varLambda ({\mathcal {A}})$ is the relative abundance of species *i* such that their total sum must be one, $\sum _{i=1}^k p_i=1.$

Note that the survey area, ${\mathcal {A}}$, will hereafter be omitted from the notation for simplicity unless it is necessary.

### The species abundance distribution

For ease of exposition in the later sections, species abundance distribution (SAD: McGill et al. 2007) is introduced here. SAD is also known as the size index (Sibuya 1993) or the frequency of frequencies (Good 1953) in more general contexts. This index represents a sequence of the number of species that occur with a certain frequency within an observed community. In the ecological literature, there has been variation in defining SAD (Shimadzu and Darnell 2015), so a concrete definition is presented here following McGill et al. (2007) who define SAD based on observations and as conditioning on a sample of *n* pooled individuals.

#### Definition 3

(*Species abundance distribution; SAD*) The number of species for which the numerical abundance, the number of individuals, is exactly *j* in *n* individuals is defined as

4 $$\begin{aligned} S_j(n) = \sum _{i=1}^k I(N_i =j | N=n), \end{aligned}$$

where $$S(n)=\sum _{j=1}^n S_j(n)$$ and $$N=\sum _{j=1}^n j S_j(n)$$.

### A link between richness and conditional richness

A key objective of ecological research is to find an underlying pattern in ecological communities rather than describing the observations of a particular occasion. This objective corresponds to investigating the expected value of species richness in the present context. The expected conditional richness is therefore needed for comprehensive discussion. Taking the expectation of SAD (4), the expected conditional richness is expressed as

5 $$\begin{aligned} {\mathbb E}_{}\left[ S(n) \right]= & {} \sum _{j=1}^n {\mathbb E}_{}\left[ S_j(n) \right] \nonumber \\= & {} \sum _{j=1}^n \sum _{i=1}^k \Pr (N_i=j | N=n)\nonumber \\= & {} \sum _{i=1}^k \Pr (N_i > 0 | N=n)\nonumber \\= & {} \sum _{i=1}^k (1- \Pr (N_i = 0 | N=n))\nonumber \\= & {} k - {\mathbb E}_{}\left[ S_0(n) \right] . \end{aligned}$$

Note that the expected SAD of the *j*th frequency is given as

6 $$\begin{aligned} {\mathbb E}_{}\left[ S_j(n) \right] = \sum _{i=1}^k {n \atopwithdelims ()j} {p_i}^j (1-p_i)^{n-j}. \end{aligned}$$

This formulation naturally follows from the multinomial distribution (Eq. 3), the marginal distribution of which is expressed as a binomial distribution.

#### Remark 1

Species richness is a function of both abundance, *n*, and evenness (relative abundance), $p_i$’s, as shown above (Eq. 6). There has been variation in the way that richness has been defined in the ecological literature, and some works define richness based on the Hill number (Hill 1973), $( \sum _{i=1}^k p_i^q )^{1/(1-q)}$, with its power coefficient being zero, $q = 0$. However, this approach does not necessarily mean that species richness is independent of evenness or abundance but is rather a formality with less ecological interpretation *per se*.

There is a clear link between richness (1) and conditional richness (2) through their expectations. They are asymptotically equivalent, *viz*.

7 $$\begin{aligned} {\mathbb E}_{}\left[ S \right] \simeq {\mathbb E}_{}\left[ S(n) \right] . \end{aligned}$$

This relationship is due to the well-known Poisson limit, applying it to Eq. (6). If the total number of individuals *n* tends to infinity and the relative abundance $p_i$ tends to zero in such a way that their product, the mean abundance of species *i*, remains constant as $n p_i = \varLambda _i$, then it becomes

8 $$\begin{aligned} \gamma _j (\varLambda _i) = \frac{{\varLambda _i}^j}{j!} e^{-\varLambda _i} \simeq {n \atopwithdelims ()j} {p_i}^j (1-p_i)^{n-j}. \end{aligned}$$

This fact leads to another useful fact about the expected SAD of the *j*th frequency (6), that is $\sum _{i=1}^k \gamma _j (\varLambda _i) = {\mathbb E}_{}\left[ S_j \right] \simeq {\mathbb E}_{}\left[ S_j(n) \right]$ and supports Eq. (7). Note that approximations (7) and (8) will frequently be used hereafter.

Further, the expected richness is, in fact, the marginal expectation of the expected conditional richness, as follows:

9 $$\begin{aligned} {\mathbb E}_{}\left[ S \right] = {\mathbb E}_{}\left[ {\mathbb E}_{}\left[ S(n) | N=n \right] \right] = k - {\mathbb E}_{}\left[ S_0 \right] . \end{aligned}$$

#### Remark 2

One desirable feature for the number of species is countable additivity. Both richness (1) and conditional richness (2) satisfy countable additivity if and only if there are no shared species amongst non-overlapped sites ${\mathcal {A}}_j (j=1, 2, \ldots , J)$, ${\mathcal {A}}_j \cap {\mathcal {A}}_{j'} = \emptyset$ for $j \ne j'$. Recalling the framework (Sect. 3.1), the richness of area ${\mathcal {A}}_j$ can be denoted by $S({\mathcal {A}}_j)$ and the feature of countable additivity is then described as ${\mathbb E}_{}\left[ S(\cup _{j=1}^J {\mathcal {A}}_j) \right] = \sum _{j=1}^J {\mathbb E}_{}\left[ S({\mathcal {A}}_j) \right] .$ For conditional richness, this feature is given as ${\mathbb E}_{}\left[ S(\sum _{j=1}^J n_j, \cup _{j=1}^J {\mathcal {A}}_j) \right] = \sum _{j=1}^J {\mathbb E}_{}\left[ S(n_j, {\mathcal {A}}_j) \right] .$

## Rarefaction techniques

The comparison of the richness observed at different sites or time points normally adopts rarefaction techniques that are formulated in one of two ways. The form of these indices can be specified in the expectation of conditional richness, ${\mathbb E}_{}\left[ S(n^*) \right]$, and the way in which the rarefied sample of $n^*$ individuals is assembled distinguishes these two techniques. One form conditions on an equalised number of individuals, and the other conditions on the same degree of sampling completeness. Note that once rarefaction is performed, the size of rarefied samples, $n^*$, no longer follows the Poisson distribution with a mean of $\varLambda$ that is discussed in Sect. 3.1, but follows an altered distribution, the mean of which is accordingly adjusted by the fraction of rarefaction.

Consider a situation comparing the richness of ecological communities at different sites or time points and in which community size differs, $n_t, t=1, 2, \ldots$. The most commonly used rarefaction technique, that based on an equalised number of individuals, is then defined as follows.

*Size-based rarefaction* The conventional rarefaction measure counts the number of species found amongst a fixed number of individuals, $n^*$, which is normally the minimum number of individuals of a community at different occasions, $n^*=\min _t \{ n_t \}$. If a pooled sample’s size $n_t$ is greater than $n^*$, the conventional rarefaction procedure draws a single subsample of $n^*$ individuals from the pooled sample of size $n_t$, and then counts the number of species found. The size-based rarefied richness of occasion *t* is then expressed as

$$\begin{aligned} R_t(n^*) = S \left( \min _t \{ n_t \} \right) . \end{aligned}$$

The rarefaction process is essentially a simple random sampling. For example, if there are *n* individuals in a pooled sample, then *rarefied* richness is calculated based on a subsample of $n^*=n-m$ individuals that are randomly selected. This approach means that the observed number of species varies depending upon the selected sample. The following proposition states the extent to which a negative bias is induced by the rarefaction process.

### Proposition 1

When the pooled sample of *n* individuals is reduced to $(n-m)$ individuals by rarefaction, the expected number of missing species is given as

10 $$\begin{aligned} {\mathbb E}_{}\left[ S(n) \right] - {\mathbb E}_{}\left[ S(n-m) \right]= & {} {\mathbb E}_{}\left[ S_0(n-m) \right] - {\mathbb E}_{}\left[ S_0(n) \right] \nonumber \\= & {} \sum _{i=1}^k \left\{ 1-(1-p_i)^m \right\} (1-p_i)^{n-m} \end{aligned}$$

where $0 < p_i \le 1$ is the relative abundance of species *i*.

### Proof

From Eq. (5), ${\mathbb E}_{}\left[ S(n) \right] - {\mathbb E}_{}\left[ S(n-m) \right] = (k-{\mathbb E}_{}\left[ S_0(n) \right] )-(k-{\mathbb E}_{}\left[ S_0(n-m) \right] )$. Substituting $j=0$ in Eq. (6) to calculate the components ${\mathbb E}_{}\left[ S_0(n) \right]$ and ${\mathbb E}_{}\left[ S_0(n-m) \right]$, the desired result follows. $\square$

### Remark 3

There is a link to another rarefaction approach proposed by Coleman (1981). Noting Eq. (7) due to the Poisson limit, Eq. (10) in Proposition 1 can be rewritten in an asymptotic form as

11 $$\begin{aligned} {\mathbb E}_{}\left[ S(n) \right] - {\mathbb E}_{}\left[ S(n-m) \right]= & {} {\mathbb E}_{}\left[ S_0(n-m) \right] - {\mathbb E}_{}\left[ S_0(n) \right] \nonumber \\\simeq & {} \sum _{i=1}^k \left( 1-e^{-m p_i} \right) e^{-(n-m) p_i}. \end{aligned}$$

This approximation (11) is satisfactory as sample size *n* is often relatively large in actual study situations.

Some useful applications of Proposition 1 can be seen in a range of ecological studies, including the following typical examples.

### Example 1

(Species accumulation curve) The species accumulation curve is a standard illustration tool that exhibits the extent to which the number of species increases as the size of the sample increases. Colwell et al. (2012) discussed the aspects of interpolation and extrapolation in the context of the species accumulation curve. Depending on which of these values, *n* or $(n-m)$, is regarded as the initial sample size, Eq. (10) can easily be converted to either interpolation (rarefaction) or extrapolation. Given a collection of *n* individuals, the expected conditional richness of a rarefied sample with $(n-m)$ individuals is expressed as interpolation,

$$\begin{aligned} {\mathbb E}_{}\left[ S(n-m) \right] = {\mathbb E}_{}\left[ S(n) \right] + {\mathbb E}_{}\left[ S_0(n) \right] - {\mathbb E}_{}\left[ S_0(n-m) \right] . \end{aligned}$$

With a sample of *n* individuals, the expected conditional richness of a sample of $(n+m)$ individuals is, on the other hand, described as extrapolation,

$$\begin{aligned} {\mathbb E}_{}\left[ S(n+m) \right] = {\mathbb E}_{}\left[ S(n) \right] + {\mathbb E}_{}\left[ S_0(n) \right] - {\mathbb E}_{}\left[ S_0(n+m) \right] . \end{aligned}$$

### Example 2

With the number of $(n-1)$ individuals identified, the probability of the next individual, the *n*th individual, belonging to one of those species already found is called *coverage*. Taking $m=1$ in Proposition 1 and using Eq. (10), the coverage becomes

$$\begin{aligned} C(n)= & {} 1 - \left( {\mathbb E}_{}\left[ S(n) \right] - {\mathbb E}_{}\left[ S(n-1) \right] \right) \\= & {} 1 - \left( {\mathbb E}_{}\left[ S_0(n-1) \right] - {\mathbb E}_{}\left[ S_0(n) \right] \right) \\= & {} 1- \sum _{i=1}^k p_i (1-p_i)^{n-1}\\= & {} 1- n^{-1}{\mathbb E}_{}\left[ S_1(n) \right] . \end{aligned}$$

Substituting the expectation, ${\mathbb E}_{}\left[ S_1(n) \right]$, with the observation, the number of species with only one individual, say, $s_1$, a simple coverage estimate is given as

12 $$\begin{aligned} \hat{C} (n, k_1) = 1- s_1 n^{-1}. \end{aligned}$$

This estimate is called the Good–Turing estimate. Chao and Shen (2010) proposed another coverage estimate based on two types of the number of species: singletons, $s_1$, and doubletons, $s_2$,

$$\begin{aligned} \hat{C} (n, s_1, s_2) = 1-\frac{s_1}{n} \left\{ \frac{(n-1)s_1}{(n-1)s_1 + 2s_2} \right\} . \end{aligned}$$

The other type of rarefaction that is discussed in the present study is formalised by Chao and Jost (2012) and applies a different approach based on coverage, rather than sample size. This approach is defined as follows.

*Coverage-based rarefaction* Given different samples of size $n_t$ with coverage $C(n_t)=q_t, t=1, 2, \ldots$, coverage-based rarefaction counts the number of species conditioning on a certain coverage, say, *q*, that yields the size of the rarefied sample as $n_t^*=\lfloor C^{-1}(q) \rfloor$. Here, $\lfloor \cdot \rfloor$ is the floor function. Chao and Jost (2012) suggest *q* to be $\min _t \{ q_t\}$. The coverage-based rarefied richness is then expressed as

$$\begin{aligned} \tilde{R}(n^*_t) = S \left( \lfloor C^{-1}(q) \rfloor \right) . \end{aligned}$$

As a consequence of this approach, the size of the rarefied sample $n_t^*$ varies, but the coverage, *q*, remains the same across places and occasions, $t=1, 2, \ldots$.

### Remark 4

If the coverage increases towards completeness, $q \rightarrow 1$, the expectation of coverage-based rarefied richness tends to the expectation of richness (Eq. 7), as

$$\begin{aligned} \lim _{q \rightarrow 1} {\mathbb E}_{}\left[ \tilde{R}(n^*_t(q)) \right] = {\mathbb E}_{}\left[ S_t \right] . \end{aligned}$$

Since $n^*_t(q)={\mathbb E}_{}\left[ S_1 (n_t) \right] /(1-q)$, adopting Eq. (12), $n^*_t(q)$ tends to infinity when *q* goes to 1. Recall the present framework in Sect. 3, by which $n_t p_{it}$ tends to $\varLambda _{it}$ (Eq. 8).

### Remark 5

Chao and Jost (2012) showed that as a unique feature of coverage-based rarefied richness, $\tilde{R}(n^*)$, this measure satisfies the property called the *replication principle*, in other words, countable additivity (Remark 2). For different non-overlapped sites ${\mathcal {A}}_j, j=1, 2, \ldots , J$, if there are no shared species amongst the sites, this measure is given as

$$\begin{aligned} \tilde{R} \left( \lfloor C^{-1}(q) \rfloor , \cup _{j=1}^J {\mathcal {A}}_j\right) = J \tilde{R} \left( \lfloor C^{-1}(q) \rfloor , {\mathcal {A}}_j\right) . \end{aligned}$$

**Table 1:** The difference between two rarefaction techniques

	Size-based	Coverage-based
Rarefied sample size ($n_t^*$)	$\min _t \{ n_t \}$	$\lfloor C^{-1}(q) \rfloor$
The range of $n_t^*$	$n^*\le n_t$	$n_t^*\le n_t$ or $n_t^* > n_t$
Time varying ($dn_t^*/dt$)	No ($=0$)	Yes

Table 1 summarises the differences between the two rarefaction techniques discussed. Note that the way determining rarefied sample size $n_t^*$ distinguishes these two techniques and treats them as simple random sampling. A key distinction is whether the rarefied sample size, the number of individuals from which the number of species is counted, varies over time $t=1, 2, \ldots$. This number remains constant for size-based rarefaction as $n^*=\min _t \{ n_t \}$ but varies for coverage-based rarefaction as $n_t^*=\lfloor C^{-1}(q) \rfloor$, where $q=\min _t \{ C(n_t)\}$. This feature becomes key when investigating the components of richness change in the next section.

## Components of richness change

For ease of exposition, the study here focuses on temporal changes in species richness that can be studied by the gradient (slope) of expected conditional richness, $d{\mathbb E}_{}\left[ S(n_t) \right] /dt$. Although this indicator is sensible for quantifying the amount of change in biodiversity over time, it has received little investigation as to whether the two different rarefaction techniques acquire the same gradient. The statements presented in this section argue that these techniques are different from each other and that they shed light on very different aspects of temporal changes in biodiversity.

Recalling Sect. 3.1, the numerical abundance, $N_{it}$, of species *i* follows a Poisson distribution: $\gamma _j (\varLambda _i) = \Pr (N_{it} = j ; \varLambda _i) = e^{-\varLambda _i} \varLambda _i^j / j!$. First, it is useful to study the extent to which the expected SAD of the *j*th frequency changes over time.

### Lemma 1

The gradient of expected SAD of the *j*th frequency with a reasonably large sample size, $n_t$, can be expressed as

13 $$\begin{aligned} \frac{d}{dt} {\mathbb E}_{}\left[ S_j(n_t) \right]\simeq & {} \sum _{i=1}^k \left( \gamma _{j-1}(n_t p_{it}) - \gamma _j (n_t p_{it}) \right) \frac{d}{dt} n_t p_{it}, \end{aligned}$$

where $p_{it}$ is the relative abundance of species *i*. Here, it is promised that $\gamma _{-1}(n_t p_{it})=0$.

### Proof

Using the Poisson limit (Eq. 8), ${\mathbb E}_{}\left[ S_j(n_t) \right] \simeq \sum _{i=1}^k \gamma _j(n_t p_{it})$. Taking the derivative of $\gamma _j(n_t p_{it})$ with respect to time *t*, the result follows. $\square$

The gradient of expected conditional richness is then expressed as follows.

### Theorem 1

The gradient of expected conditional richness, conditioning on a sample of reasonably large size, $n_t$, is given as

14 $$\begin{aligned} \frac{d}{dt} {\mathbb E}_{}\left[ S(n_t) \right]\simeq & {} \sum _{i=1}^k \gamma _1(n_t p_{it}) \frac{d}{dt} \log (n_t p_{it}), \end{aligned}$$

where $\gamma _1(n_t p_{it})$ is the probability of species *i* to be singleton, and $p_{it}$ is the relative abundance of the species.

### Proof

Using Eq. (13) in Lemma 1 and the Poisson limit (8),

$$\begin{aligned} \frac{d}{dt} {\mathbb E}_{}\left[ S(n_t) \right]\simeq & {} \sum _{j=1}^\infty \frac{d}{dt} {\mathbb E}_{}\left[ S_j(n_t) \right] \\= & {} \sum _{i=1}^k \sum _{j=1}^\infty \left( \gamma _{j-1}(n_t p_{it}) - \gamma _j (n_t p_{it}) \right) \frac{d}{dt} n_t p_{it}\\= & {} \sum _{i=1}^k \left\{ 1-\left( 1-\gamma _{0}(n_t p_{it}) \right) \right\} \frac{d}{dt} n_t p_{it}, \end{aligned}$$

and the desired result follows. Note that the toal mass probability of a Poission distribution is $\sum _{j=0}^\infty \gamma _j (n_t p_{it})=1$. $\square$

Expanding the right-hand side in Eq. (14) further, Theorem 1 clearly highlights the fact that the temporal change in species richness is formed of two components in an additive manner, *viz*.

15 $$\begin{aligned} \frac{d}{dt} {\mathbb E}_{}\left[ S(n_t) \right] \simeq {\mathbb E}_{}\left[ S_1(n_t) \right] \frac{d}{dt} \log \left( n_t\right) + \sum _{i=1}^k \gamma _1 (n_t p_{it}) \frac{d}{dt} \log \left( p_{it} \right) . \end{aligned}$$

These two terms on the right-hand side in Eq. (15) are directly linked with the temporal changes in community abundance, $n_t$, and species relative abundance, $p_{it}$, that are two key components in quantitative ecology (Brown 1981). An equivalent decomposition for the expectation of numerical abundance, the number of individuals, was also derived in Shimadzu et al. (2015), in which the temporal turnover of ecological communities was discussed.

In biodiversity research, the comparison of species richness is performed by comparing rarefied richness, which has a sample size $n_t^*$, instead of the conditional richness, the sample size of which is $n_t$. Equation (14) in Theorem 1 states that the gradient of rarefied richness can also be calculated by substituting $n_t$ with $n^*_t$ as the general rarefaction context.

For the cases of the two rarefied richness indicators, a clear distinction can be summarised in the following corollaries.

### Corollary 1

Given a rarefied sample of size $n^* = \min _t \{ n_t\} = n_t - m_t$, the gradient of expected size-based rarefied richness is given as

16 $$\begin{aligned} \frac{d}{dt} {\mathbb E}_{}\left[ R_t(n^*) \right] \simeq \sum _{i=1}^k w_{it} \gamma _1(n_t p_{it}) \frac{d}{dt} \log (p_{it}), \end{aligned}$$

where $$w_{it} = \gamma _0 (m_t/n_t - m_t p_{it})$$ for $$m_t \ll n_t$$.

### Proof

From Eq. (14) in Theorem 1

$$\begin{aligned} \frac{d}{dt} {\mathbb E}_{}\left[ R_t(n^*) \right] \simeq \sum _{i=1}^k \gamma _1(n^*_t p_{it}) \frac{d}{dt} \log (n^*_t p_{it}). \end{aligned}$$

Here, $\gamma _1(n^*_t p_{it})$ can be rewritten as

$$\begin{aligned} \gamma _1 (n^*_t p_{it})= & {} n^*_t p_{it} \gamma _0 (n^*_t p_{it}) = (n_t-m_t) p_{it} \gamma _0 ((n_t-m_t) p_{it}) \\= & {} (n_t-m_t) p_{it} \gamma _0 (-m_t p_{it}) \gamma _0 (n_t p_{it}) \\= & {} (1- m_t/n_t) \gamma _0 (-m_t p_{it}) \gamma _1 (n_t p_{it})\\\simeq & {} \gamma _0\left( m_t/n_t -m_tp_{it} \right) \gamma _1 (n_t p_{it}). \end{aligned}$$

Noting the fact that $d\log (n^*_t)/dt=0$ as $n^*_t=\min _t \{ n_t\}$, the desired result follows.

$$\square $$

This result (Eq. 16) reveals that the trend of the size-based rarefied richness reflects only the change in community composition, $p_{it}$’s, suggesting a link to an information approach (Margalef 1957, 1958) that has commonly been used in biodiversity research and also relies only on the community composition. If the term omitted from Eq. (15), which is related to the change in community size $n_t$, plays a key role, the calculated gradient is not able to represent the trend of species richness. However, whether using this approach is appropriate largely depends upon the scientific question asked.

### Corollary 2

Suppose a rarefied sample of size $n_t^*= {\mathbb E}_{}\left[ S_1(n_t) \right] /(1-q)$ for an arbitrary coverage $0 < q \le 1$. The gradient of expected coverage-based rarefaction is given as

17 $$\begin{aligned} \frac{d}{dt} {\mathbb E}_{}\left[ \tilde{R}(n_t^*) \right] \simeq \tilde{w}_{1t} \frac{d}{dt} {\mathbb E}_{}\left[ S_1(n_t) \right] +\frac{d}{dt} {\mathbb E}_{}\left[ R_t(n_t^*) \right] , \end{aligned}$$

where $\tilde{w}_{1t} = {\mathbb E}_{}\left[ S_1(n_t^*) \right] /{\mathbb E}_{}\left[ S_1(n_t) \right]$ is a weight due to rarefaction.

### Proof

Since $n^*_t ={\mathbb E}_{}\left[ S_1(n_t) \right] /(1-q)$, using Eq. (14) in Theorem 1, it becomes

$$\begin{aligned} \frac{d}{dt} {\mathbb E}_{}\left[ \tilde{R}(n_t^*) \right]\simeq & {} \sum _{i=1}^k \gamma _1(n^*_t p_{it}) \frac{d}{dt} \left( \log \left( \frac{{\mathbb E}_{}\left[ S_1(n_t) \right] }{1-q} \right) + \log (p_{it}) \right) \\= & {} \frac{{\mathbb E}_{}\left[ S_1(n_t^*) \right] }{{\mathbb E}_{}\left[ S_1(n_t) \right] } \frac{d}{dt} {\mathbb E}_{}\left[ S_1(n_t) \right] + \sum _{i=1}^k \gamma _1(n^*_t p_{it}) \frac{d}{dt} \log (p_{it}). \end{aligned}$$

Noting Corollary 1, the result then follows. $\square$

Corollary 2 above suggests that the gradient of coverage-based rarefied richness also consists of two components, the changes in community size $n_t$ and composition $p_{it}$’s. To highlight the relationship to the expected gradient of species richness (14), the first term on the right-hand side in Eq. (17) can be expanded as

$$\begin{aligned} \tilde{w}_{1t} \frac{d}{dt} {\mathbb E}_{}\left[ S_1(n_t) \right] = \tilde{w}_{1t} \frac{d}{dt} \left\{ {\mathbb E}_{}\left[ S(n_t) \right] + \left( {\mathbb E}_{}\left[ S_0(n_t) \right] + {\mathbb E}_{}\left[ S_1(n_t) \right] \right) \right\} . \end{aligned}$$

This expansion is due to Eq. (13) in Lemma 1, in other words, due to the fact that $d({\mathbb E}_{}\left[ S(n_t) \right] +{\mathbb E}_{}\left[ S_0(n_t) \right] )dt = dk/dt = 0$ (Eq. 5). This fact highlights that the expected gradient of coverage-based richness is linearly related to that of species richness (14). If the two gradients $d{\mathbb E}_{}\left[ S_0(n_t) \right] /dt$ and $d{\mathbb E}_{}\left[ S_1(n_t) \right] /dt$ are offsetting each other, the gradient of expected coverage-based rarefaction (17) can be a reasonable proxy of species richness (14).

## An illustrative example

Here, a simulation study exhibits the extent to which the difference in temporal richness change is exposed when the two different rarefaction techniques are employed. The ecological community to be considered here is a species assemblage dominated by low abundance species, a typical community in real-world situations, that has often been reported in the ecological literature (Magurran and Henderson 2003).

What the majority of species with low relative abundance $p_{it}$ imply is that these species will demonstrate fairly low temporal change in the relative abundance, $dp_{it}/dt$. For this scenario, the theory discussed in the previous section predicts that the size-based rarefaction approach can yield a flat gradient (Corollary 1) which will be different from that of the coverage-based rarefaction approach accounting for the change in community size as well (Corollary 2).

The following simulation result confirms the theory presented and illustrates the situation where the temporal trends of size- and coverage-based approaches reach different conclusions that are somewhat contradictory. This case can be typical in practical situations where extra care is required when interpreting the results of richness change analyses.

### Simulation setup

Recall the settings in Sect. 3.1. Consider an ecological community potentially consisting of 100 species ($k=100$), the large majority of which exhibit low abundance so that the mean abundance is less than one, $\varLambda _{it}<1$. Note that such a species composition reflects reality well, resembling patterns commonly observed in field studies (Dornelas 2010). The community at time *t* is then described as a vector: $\varvec{N}_t =(N_{1t}, N_{2t}, \ldots , N_{100t})$, where $N_{it}$ is the number of individuals, numerical abundance of species *i*, governed by a Poisson distribution, that is $N_{it} \sim \mathrm{Poiss}(\varLambda _{it})$. For ease of exposition, the mean abundance of species *i* is assumed to increase or decrease linearly over time with rate $r_i$ such that $\varLambda _{it} = {\mathbb E}_{}\left[ N_{it} \right] = \max \{0, \varLambda _{i0} + r_i t \}$. A normal-distributed random number is allocated to each species’ change rate, $r_i$, so that $r_i \sim {\mathcal {N}}(-0.7, 1)$, but it remains constant over time.

The simulation was run 1000 times ($\ell =1, 2, \ldots , 1000$), generating the collection of the number of individuals, $\varvec{N}_t=\varvec{n}^{[\ell ]}_t$, for 100 time steps ($t=1, 2, \ldots ,100$). For each run, the two types of rarefaction measure were calculated. One measure was size-based rarefaction, the conventional approach, which has a rarefied sample size $n^{*[\ell ]} = \min _t \{n^{[\ell ]}_t \}$; the other was the coverage-based rarefaction measure, which has a rarefied sample size $n_t^{*[\ell ]} = \lfloor C^{-1}(q^{[\ell ]}) \rfloor$, where $q^{[\ell ]}=\min _t \{C(n^{[\ell ]}_t) \}$. The rarefaction process followed as described in Sect. 4. At each simulation step, a linear trend was calculated for each rarefied richness series: size-based $R_t$ and coverage-based $\tilde{R}_t$.

### Results

**Fig. 1:**
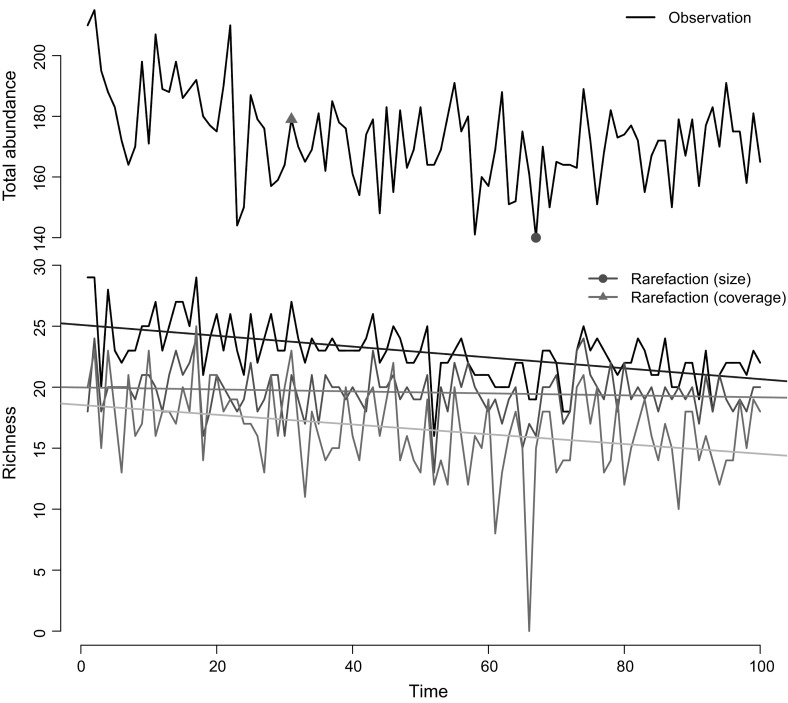
A snapshot, one of the 1000 simulations run. Top: An observed series of total abundance simulated from the assumed system consisting of 100 species of which abundance follows a Poisson distribution. The red dot on the line is the minimum number of individuals, the rarefied sample size to which all the size-based rarefied samples are equalised. The magenta triangle is the observation point that achieved the minimum converge was achieved and to which all the coverage-based rarefied samples are adjusted. Bottom: The solid black line is observed richness. The red line represents size-based rarefied richness and the purple line represents coverage-based rarefied richness. The three superposed straight lines are the linear trend of each richness series over time (color figure online)

Figure 1 shows a snapshot, one of the 1000 simulations run, as an illustration. For each simulation step, an ecological community is generated over 100 time steps, $\varvec{N}_t =\varvec{n}^{[\ell ]}_t, (t=1, 2, \ldots , 100)$ (Fig. 1, top panel). The overall abundance of the community decreases over time. Three types of richness are acquired from the community (Fig. 1, bottom panel). The solid black line represents the conditional richness $S(n_t)$ series, in other words, the observed richness; the solid red and magenta lines are the two types rarefied richness: size-based $R_t(n^*)$ and coverage-based $\tilde{R}(n_t^*)$. The three superimposed straight lines (blue, green and cyan) are the temporal linear trend of each species richness series. The lines for conditional (blue) and coverage-based rarefied richness (cyan) both illustrate a similar decreasing pattern, although the line for size-based rarefied richness (green) suggests a flat trend.

**Fig. 2:**
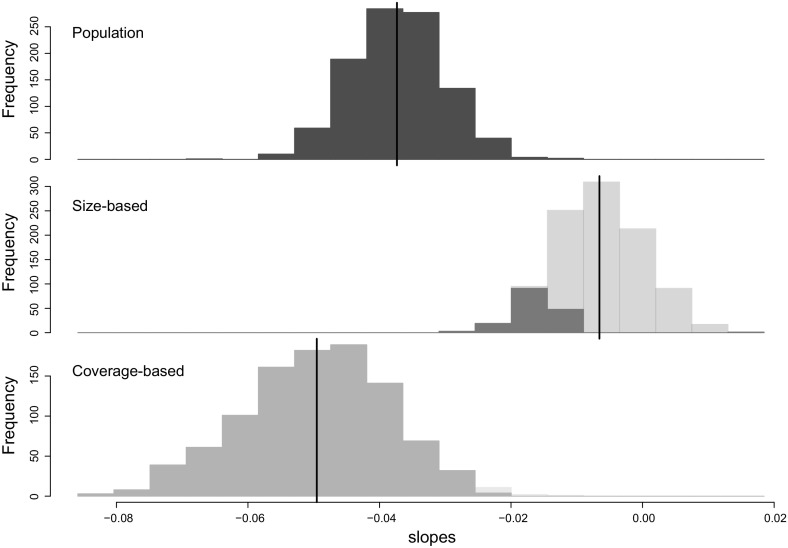
The histograms of the slope coefficients from the 1000 simulation study. The blue one (top) represents the distribution of the slope coefficients of the observed richness series. The green (middle) and the cyan (bottom) histograms show the distribution of the slope coefficients of size-based rarefied richness and of coverage-based rarefied richness, respectively. The transparent colour highlights statistically non-significant coefficients, under the null hypothesis in which the slope coefficient is zero. The black vertical lines are their averages (color figure online)

The pattern observed in Fig. 1 was, in fact, persistent over the 1000 simulations. Figure 2 summarises the distribution of the gradient (slope coefficient) of the linear trend over the 1000 simulations for each richness measure. This figure suggests that the size-based rarefaction technique tends to mute the decline of species richness (Fig. 2, middle panel) as the green histogram is shifted towards zero relative to the blue histogram, illustrating the observed richness (Fig. 2, top panel). Specifically, if the trend of the size-based rarefaction is judged as to whether it departs considerably from a flat line, $85 \%$ of the coefficients are classified as statistically non-significant—the transparent green area in the middle panel of Fig. 2. In contrast, the cyan histogram for the coverage-based rarefaction measure indicates a negative trend in species richness (Fig. 2, bottom panel), as does the observed richness. This typical illustration underlines the importance of clarifying which aspect of biodiversity change is being sought when using species richness as an indicator. Otherwise, what the increasing or decreasing trend means becomes unclear.

## Discussion and concluding remarks

The present study has revealed that the analysis of richness change can delineate the different aspects of biodiversity change depending on the types of rarefaction technique employed; in other words, the result depends upon the sampling fraction induced by the rarefaction. This finding indicates the fact that the analysis of richness likely draws different, sometimes contradictory, conclusions if different rarefaction techniques are adopted. The size-based rarefied richness indicator reflects mainly the change in species composition, $p_{it}$’s, (Corollary 1), whereas the coverage-based rarefaction indicator presents the changes in both components: species composition, $p_{it}$’s, and community size, $n_t$, (Corollary 2). The latter rarefaction technique may better resemble the slope of species richness (Fig. 1) but relies heavily on the construction of species composition. This fact, however, seems to have rarely been acknowledged amongst researchers. The new insights provided here suggest that extra care is therefore required when interpreting changes in species richness as the choice of rarefaction method will drive the results.

Which rarefaction technique is adequate largely depends upon the scientific question asked. Although the coverage-based rarefied richness indicator is able to accommodate the changes in both community size and composition, this feature does not necessarily mean that the approach surpasses the size-based method. For instance, if the question asked focuses on changes in community composition, investigating richness changes using size-based rarefaction will yield a more realistic figure than the coverage-based method.

The compositional change of ecological communities has historically been a central question in biodiversity research, and a number of ecological theories stand directly on a base assumption that community size is constant (Gotelli et al. 2017). This subject is related to a recent argument raised and discussed by Hillebrand et al. (2017) indicating that species richness is insensitive in quantifying the change in biodiversity compared to a set of turnover indices. The change in species richness additively consists of the two components: the changes in community size and composition. If the shift in community size is considerable in terms of the fraction of the change, the compositional change is then relatively muted. This offsetting is the mechanism that makes richness measures occasionally insensitive to biodiversity change. However, this insensitivity underlines the importance of clarifying what aspects of biodiversity change are sought rather than just choosing one of the commonly used biodiversity indices.

The framework presented in the study can also be discussed in a more general context concerning sampling effects in ecological data. A range of sampling methods used for collecting ecological data can be specified as types of random sampling (Shimadzu et al. 2016). The rarefaction techniques discussed here are, in fact, specified as simple random sampling with the fraction of rarefaction. Another typical source of uncertainty in ecological surveys can also be treated in the same manner by regarding the process as two-step sampling procedure: sampling and rarefaction. See Shimadzu et al. (2016) for more details. For a general description of sampling processes in ecological surveys, a thinned Poisson point process is a handy model (Cressie 1993; Illian et al. 2008).

The present change in biodiversity underpins the future patterns of biodiversity. This fact illuminates the importance of understanding what aspects of biodiversity change the commonly used biodiversity indices are able to capture. In particular, for species richness, the theoretical framework developed in the present study has uncovered a key fact that different rarefaction techniques highlight different aspects of biodiversity change. The analysis results can, therefore, be different depending on the rarefaction method used. Nevertheless, such a consequence has received little attention. The study argues that the recognition of the statistical nature of biodiversity indices is a crucial step towards enhancing the knowledge of biodiversity change at a time when these changes are becoming a great matter in society.
